# Oxidative Stress in the Poultry Gut: Potential Challenges and Interventions

**DOI:** 10.3389/fvets.2019.00060

**Published:** 2019-03-04

**Authors:** Birendra Mishra, Rajesh Jha

**Affiliations:** Department of Human Nutrition, Food and Animal Sciences, College of Tropical Agriculture and Human Resources, University of Hawaii at Manoa, Honolulu, HI, United States

**Keywords:** oxidative stress, gastrointestinal tract, antioxidant, poultry production, heat stress

## Abstract

The gastrointestinal tract (GIT) provides the biological environment for nutrient digestion and absorption, and protection from pathogens and toxins. Broilers are fast growing because of the great potential of intestinal epithelia for nutrient absorption, and efficient conversion of nutrient to muscle. Physiologically, reactive oxygen species (ROS) and reactive nitrogen species (RNS) are generated by GIT epithelial cells either from oxygen metabolism or by enteric commensal bacteria and regulate gut health. However, increased production of ROS elevates free radical production and antioxidant insults resulting in oxidative stress. Oxidative stress in poultry GIT is derived from nutritional, environmental heat stress, and pathological factors, which alters overall performance as well as meat and egg quality. Supplementation of exogenous vitamins, antioxidants, and plant extract having antioxidant properties scavenge ROS and are beneficial in mitigating oxidative stress in the GIT. This review highlights the involvement of oxidative stress in the gastrointestinal functionality of poultry and potential intervention strategies to maintain redox balance in the GIT.

## Introduction

Poultry is one of the fastest growing animal industry and has a substantial contribution to food security and nutrition. Poultry meat and eggs are among the most common animal source of food consumed at the global level. The poultry and egg industries are among the largest agricultural commodities globally. Over the period, immense improvements have been made in genetics, feed conversion ratio, fat reduction, and breast size of broiler chickens and significant improvement in the hen-day egg production and egg quality in laying hen (1, 2). In the poultry industry, the feed is the major component of the total cost for meat and egg production. Also, feed exposes the birds to a wide variety of factors through the gastrointestinal tract (GIT), and affect poultry health and production.

The GIT is a highly complex and dynamic organ, which plays a critical role in nutrient absorption and immune response (3). Intestinal mucosa, a site for nutrient absorption, is composed of heterogeneous cell populations, epithelial cells, and connective tissues. The intestinal epithelia are exposed continuously to a wide variety of potentially harmful substances and act as a selective barrier between the tissues and luminal environment of the GIT. There are several stressors such as feed toxin; infectious agents induce the cellular free radicals' generation results in redox imbalance. This stress can negatively affect the delicate balance among the components of the chicken GIT, which in turn, affect the health status and productivity of poultry. The purpose of this review is to provide updated information on different oxidative stressors, to elucidate the impact of oxidative stress on the pathophysiology of poultry GIT and potential interventions to mitigate the effects.

## Oxidative Stress

Stresses in commercial poultry result from environmental, nutritional, microbiological, and management factors which negatively impact poultry health and production. Oxidative stress is downstream of all these stresses. Oxidative stress in the cells/tissues results from an imbalance between free radical production and endogenous antioxidant defense and leads to lipid peroxidation, protein nitration, DNA damage, and apoptosis. Cells are exposed continuously with the free radicals generated during the physiological oxygen metabolism (4). Both reactive oxygen species (ROS) and reactive nitrogen species (RNS) at certain levels are signaling molecules involved in homeostasis. However, excessive production of ROS and RNS or their inefficient scavenging leads to oxidative stress. ROS, including superoxide, hydrogen peroxide, and the hydroxyl radical radicals, are generated by oxygen metabolism and further balanced by the rate of oxidant formation and the rate of oxidant elimination. The intracellular reduction of ROS is physiologically scavenged by superoxide dismutase, catalase, and glutathione peroxidase (GPX) (5). Superoxide dismutase (SOD1 and SOD2) catalyze the dismutation of the superoxide anion (${\text{O}}_{2}^{-}$) to H_2_O_2_ (6), which in turn, is decomposed into H_2_O and O_2_ by catalase, while GPX reduces lipid hydroperoxides by incorporating glutathione (6). The RNS that are by-products of nitric oxide synthases (NOS) are expressed in selected cells of the intestinal mucosa and submucosal regions. The NOS metabolizes arginine to citrulline and forms the nitric oxide radical (NO∙) which is crucial for cellular function including neurotransmission and immunomodulation. However, overproduction of nitric oxide radicals' damages intestinal mucous membrane and impaired nutrient utilization (7). Both ROS and RNS can contribute to lipid peroxidation especially cell membrane lipids and lipoproteins since they are rich in polyunsaturated fatty acids. The end product of lipid peroxidation is 4-hydroxynonenal, which increases oxidative damage to the cell membrane and impair the cell signaling and mitochondrial dysfunctions. Inflammation in GIT is mediated through several stressors/infections which in turn generate ROS and disrupt redox balance.

## Oxidative Stress Associated With Environmental Heat Stress

The high temperature is one of the most challenging environmental stressors associated with poultry production (8). Heat stress is a major source of systemic oxidative stress since it causes a redox imbalance between the pro- and anti-oxidants in favor of prooxidants. Heat stress has been shown to alter the feed intake, poor growth performance, immunosuppression, hypoxia, and high mortality (9, 10). Heat stress also deteriorates the meat quality of chicken (11). Birds under cyclic heat stress display less crypt depth, mucous area, and villus height of small intestine (12), leading to negative impact on nutrient absorption. Also, heat stress causes intestinal epithelial cell injury, and apoptosis contributes to intestinal hyperpermeability which causes the influx of bacterial products from the intestinal lumen into the circulatory system and affects organ systems. The ROS components such as superoxide anions, hydrogen peroxide, and hydroxyl radicals are produced in the mitochondria and act as signaling intermediates (5). Under physiological conditions, generated antioxidant enzymes rapidly eliminate ROS. Several studies link oxidative stress with heat stress or lipopolysaccharide (LPS), and suggest synergistic augmenting of cell death and increased ROS generation in specific cells (13). Heat stress also activates the chicken hypothalamus, pituitary adrenal axis, resulting in elevated serum corticosterone, which in turn decreases food intake, body weight gain, relative immune organ weight, and innate immunity. This neuroimmune dysfunction further alters intestinal-immune barrier, allowing pathogenic bacteria to migrate through the intestinal mucosa and generating an inflammatory infiltrate. Inflammation of the intestine also decreases nutrition absorption and consequently decrease in weight gain (14).

## Oxidative Stress Associated With Feed Toxins

Poultry feeds/feed ingredients are often contaminated with a wide range of environmental toxicants, bacterial and fungal toxins, and known to affect the gut health. The intestinal luminal epithelial cells and the tight junction proteins between two adjacent epithelial cells from the barrier and thus preventing paracellular absorption of toxins. Oxidative stress alters not only the cellular processes but also the intestinal barrier function. Mycotoxins are metabolites produced in a strain-specific way by a wide range of fungi, particularly molds. The common mycotoxins are aflatoxin, zearalenone, deoxynivalenol, trichothecenes, fumonisin, T-2 toxin, and ochratoxin. Once these toxins come in contact with the epithelial cells or during the absorption, the GIT is greatly impacted by the induction of oxidative stress. It has been shown that the chronic, long-term exposure to even low levels of mycotoxins may impact the immune system and intestinal integrity and compromise the blood phagocytic activity in chickens (15). Trichothecenes are a group of mycotoxins (deoxynivalenol, T-2 toxin, and fumonisin B1) which are mainly produced by fungi of the genus Fusarium. These mycotoxins generate ROS which induces lipid peroxidation, alters the cellular redox signaling, antioxidant status, and membrane integrity of the cells. Trichothecenes also increase the intestinal epithelial permeability. Together, mycotoxins increase cellular apoptosis and affect poultry health and production.

Arsenic is widely distributed in water, food, and the environment. It is highly toxic and causes adverse effects on digestion and absorption of nutrients, resulting in potential losses to poultry growth. Heavy or chronic arsenic exposure induces lipid peroxidation, decreases antioxidants, and eventually trigger apoptosis in several body tissues of poultry (14). Copper, arsenic, and their combination induce the inflammation and the destruction of the intestinal mucosa (16).

Ammonia is one of the primary sources of the air contaminant in the poorly ventilated poultry house. High levels of ammonia decrease growth rate, body weight, and alter feed efficiency (17). Longer exposure to ammonia also causes several health issues and compromise the welfare of broilers (18). The absorption capacity of the intestine depends on the number and size of villi. Chicken exposed to high concentration of ammonia has much lower villus height and crypt depth among different segments of the small intestine. Ammonia also exerts negative impacts on immune organ development of chickens, which may cause enormous damages to nutrient absorption and immune system (18). It has also been reported that ammonia exposure increases the activity of creatine kinase and decreased activity of serum T-SOD producing oxidative stress and apoptosis of mucosal structure (19).

## Oxidative Stress Associated With Microorganisms

In poultry production, intestinal health and function play a critical role in efficient feed utilization and growth, and the overall profitability of the farm. The GIT microbiota mainly consists of bacteria, fungi, and protozoa. Microbiota population varies across the compartment with maximal at the distal segments of the GIT (20). Intestinal epithelial in response to commensal bacteria generate ROS, which serves as a second messenger and participates in cellular signaling. Tight junctions between intestinal epithelial cells from the barrier and prevent the invasion of the microorganism (21, 22). Studies have suggested that interaction of mucosa with microbes or their toxin triggers oxidative stress. Coccidiosis is among the most common parasitic diseases of poultry. Eimeria primarily produces oxidative stress, thereby destroy the intestinal epithelial barrier and tight junctions, lipid peroxidation, antioxidants insult, as a result, infected birds display reduced feed intake, absorption of nutrients and decreases weight gains (23). Environmental heat stress affects the intestinal epithelial cells and further stimulate intestinal bacteria and bacterial LPS. The LPS is known to induce apoptosis and injury in various cell types (24).

## Anti-oxidative Systems in GIT

The intestinal mucosa is responsible for the absorption of nutrient, and the antioxidant system maintains diverse microbiota in the luminal epithelia. The intestinal mucosa is directly exposed to both feed and non-feed substances. Above physiological level, production of ROS/RNS results in intestinal inflammation and impair the absorption capacity. It has been reported that the broilers fed with the oxidized oils/fats imbalance antioxidants and immune response within the intestinal mucosa (25). As the first line of defense against oxidative stress, the intestinal mucosa contains an extensive antioxidants defense system including enzymes (CAT, SOD, or GPX) and non-enzymatic endo- and exo-genous scavengers like glutathione, transient ions (e.g., Fe2^+^, Cu2^+^) or flavonoids (26). Glutathione and SOD are intracellular antioxidants, widely distributed in the small intestine, and their abundances are at a higher level during intestinal development (27).

## Mitigation of Oxidative Stress

The feeds intake, digestion, and subsequent absorption of nutrients in the intestine produce free radicals and imbalance antioxidant system in the intestinal mucosa resulting oxidative stress (28). Also, oxidative stress damages to the intestinal mucosa impede the efficient digestion and absorption of nutrients and adversely influences normal animal growth (29). Dietary inclusion of antioxidant compounds reduces intestinal free radicals, and also help in maintaining the intestinal mucosa. Several studies have suggested that oxidative stress predisposes the birds to various pathological and welfare situation. Therefore, it is essential to formulate a cost-effective strategy to mitigate oxidative stress. Supplementations of vitamin C and E, improve antioxidant ability and immune performance (30). Alpha lipoic acid, possesses both fat and water soluble, is a potent antioxidant and is protective against oxidative damages in the poultry intestine (31). The inclusion of polyphenol compounds also exhibits potent antioxidant activity (32). Equol which is derived from the isoflavonoid daidzein, a major isoflavone of soybean, can hinder oxidative modification induced by ROS (33). Equol protect protects intestinal epithelial cells from oxidative damage by promoting the expression of antioxidant genes, increasing the activities of antioxidant enzymes, and by enhancing antioxidant capacity (34). Dietary galacto-oligosaccharides, as a prebiotic, stabilizes intestinal integrity and prevent against oxidative damages (35). The supplementation of antioxidant-containing plants extracts such as Tulbaghia violacea is shown to have a beneficial effect on the rate of Eimeria oocyst shedding (23). Dietary supplementation of L-glutamine also prevents Necrotic enteritis in antibiotic-free diets (36). Dietary glutamate and N-acetylcysteine induce several antioxidant genes and inflammatory biomarkers in the intestinal mucosa which alleviate LPS-induced intestinal inflammation (37) and can be potentially be used in the chicken diet. Therefore, based on the stress, an individual ingredient or in combination can be used to mitigate oxidative stress in the GIT.

## Conclusion

Oxidative stress in the poultry GIT is produced by the nutritional factors, environmental factors like heat stress, and pathological factors. These stresses have a negative impact on broiler growth and production as well as the quality of meat and egg produced. Currently, different antioxidants like exogenous vitamins, antioxidants, and plant extract are used individually or in combination to prevent the oxidative stress in poultry. These anti-oxidants scavenge the reactive oxygen species and reactive nitrogen species to mitigating oxidative stress in the GIT at varying level. Further studies are required to investigate the effects of antioxidants in different combination to mitigate oxidative stress in broiler chicken.

## Author Contributions

BM reviewed the literature and drafted the manuscript. RJ reviewed the manuscript and provided critical review and suggestion and comments.

### Conflict of Interest Statement

The authors declare that the research was conducted in the absence of any commercial or financial relationships that could be construed as a potential conflict of interest.
